# The serum levels of activin A and bone morphogenetic protein-4 and -6 in patients with fibrodysplasia ossificans progressiva

**DOI:** 10.1186/s13023-023-02708-3

**Published:** 2023-05-10

**Authors:** Zhengqin Ye, Siyi Wang, Chang Shan, Qi Zhu, Ying Xue, Keqin Zhang

**Affiliations:** 1 Department of Endocrinology and Metabolism, Tongji Hospital, School of Medicine, Tongji University, No. 389, Xincun Road, Shanghai, 200065 China; 2 Medical School of Nantong University, Affiliated Hospital of Nantong University, 19 Qixiu Road, Nantong, Jiangsu, China; 3 Institute of Osteoporosis and Metabolic Bone Diseases, School of Medicine, Tongji University, No. 389, Xincun Road, Shanghai, 200065 China

**Keywords:** Fibrodysplasia ossificans progressiva (FOP), Activin A, Bone morphogenetic protein 4 (BMP4), Bone morphogenetic protein 6 (BMP6)

## Abstract

**Background:**

Fibrodysplasia ossificans progressiva (FOP) is an ultrarare and disabling genetic disorder of connective tissue characterized by congenital malformation of the great toes, and progressive heterotopic ossification (HO) in soft connective tissues. A gain-of-function mutation of activin A receptor type I (ACVR1) enables ACVR1 to recognize activin A as an agonist with bone morphogenetic protein (BMP) signalling that leads to HO. Previous studies confirmed that activin A stimulates BMP signalling in vitro and drives HO in mouse models of FOP. However, the roles for BMP4 and BMP6 in FOP are supported only by correlative evidence in vitro. Thus, it remains unclear whether the circulating levels of activin A, BMP4 and BMP6 correlate with flare-ups in FOP patients. Hence, we investigated the protein levels of activin A, BMP4 and BMP6 in the serum of FOP patients.

**Results:**

We recruited 16 untreated FOP patients and 16 age- and sex- matched healthy control subjects in this study. The 16 FOP patients were retrospectively divided into the flare-up group (*n* = 8) and remission group (*n* = 8) depending on whether they had flare-ups or worsening of any joint movement in the last 6 months. The serum activin A, BMP4 and BMP6 levels were detected by enzyme-linked immunosorbent assay. The serum activin A, BMP4 and BMP6 levels were slightly higher in FOP patients (median: 434.05 pg/mL, 459.48 pg/mL and 67.84 pg/mL) versus healthy control subjects (median: 364.14 pg/mL, 450.39 pg/mL and 55.36 pg/mL). However, there were no statistically significant differences between the two groups (*p* > 0.05 for all items), nor were there significant differences between the flare-up and remission groups of FOP (*p* > 0.05 for all items). Univariate and multivariate logistic regression analyses showed that age, sex, and serum activin A, BMP4 and BMP6 levels were not related to flare-up in FOP patients.

**Conclusions:**

There were no significant differences in the serum levels of activin A, BMP4 and BMP6 in FOP patients compared with healthy control subjects. Serum activin A, BMP4 and BMP6 proteins might not be the stimulators for FOP flare-up, and may not be biomarkers for FOP diagnosis.

## Background

Fibrodysplasia ossificans progressiva (FOP) is an ultrarare and disabling genetic disorder of connective tissue that is characterized by congenital malformation of the great toes and progressive endochondral heterotopic ossification (HO) in soft connective tissues, such as skeletal muscles, tendons and ligaments, spontaneously or after minor trauma or viral infections [1, 2]. The prevalence of this disorder is approximately 1 in 2 million worldwide, 1.36 per million in France, and 0.88 per million in the United States [3–5]. We previously estimated that there are 600–700 patients with this condition in mainland China [6]. To date, we have recruited more than 130 FOP patients to our hospital. A common activating mutation in the gene encoding activin receptor IA (*ACVR1*) /activin receptor-like kinase 2 (*ALK2*), which is a bone morphogenetic protein (BMP)-type I receptor, exists in all familial and sporadic cases with a classic clinical presentation of FOP. Approximately 97% of individuals with FOP carry a gain-of-function mutation (c.617G > A; R206H) in the glycine-serine domain of ACVR1 [7], which results in enhanced BMP pathway signalling [8]. Activin A, a hormone-like factor not previously thought to play a role in this disease, is regarded as the main causative factor for FOP due to the following key facts: (1) endochondral HO could be triggered by activin A administration in the mouse model of FOP [9–11]; and (2) the inhibition of activin A with a blocking antibody completely inhibited the development of HO at the phases of both HO initiation and expansion in the FOP mouse model [9, 12]. The above evidence indicates that the role of activin A as the initiator of HO in FOP is solidly established. In addition, a recent study showed that there is a high level of transforming growth factor beta (TGF-β) signalling in the fibroblasts of FOP patients [13]. Inhibition of TGF-β signalling can decrease osteogenic differentiation of FOP in vitro [13]. Systemic administration of TGF-β neutralizing antibody could effectively inhibit HO in a mouse model of FOP [14]. These results imply that the whole developmental process (inflammatory, fibroproliferative, chondrogenic and osteogenic phases [8]) of FOP lesions is not entirely controlled by activin A, and some other factors might participate in the pathogenesis of FOP.

The expression of some bone morphogenetic proteins (BMPs) might correlate with FOP. As the infection of Epstein‒Barr virus could induce flare-ups of FOP, Shafritz et al. established lymphoblastoid cell lines from peripheral blood mononuclear cells (PBMCs) of FOP individuals and normal subjects through the transformation of the cells by Epstein‒Barr virus [15]. They found that the mRNA expression of bone morphogenetic protein 4 (BMP4) was positive in 81% of the lymphoblastoid cell lines from FOP patients and positive only in 8% of normal control cell lines (*p* < 0.01). These findings suggest that the overexpression of BMP4 in lymphocytes might correlate with FOP. In addition, both primary connective tissue progenitor cells and induced human pluripotent stem cell-derived endothelial cells from FOP patients showed increased SMAD1/5/8 signalling upon BMP4 stimulation [16, 17], and SMAD1/5/8 is the downstream protein complex of ACVR1^R206H^. We found by transcriptome analysis that the bone morphogenetic protein 6 (BMP6) mRNA content in PBMCs of FOP subjects was significantly higher than that of healthy control subjects (data not shown). In vitro data indicated that BMP6 enhanced SMAD1/5 phosphorylation in fibroblast cells from FOP patients [18]. It has also been reported that ACVR1^R206H^ shows a higher sensitivity towards BMP6 than ACVR1^WT^ does in vitro [19]. Furthermore, BMP4 and BMP6 are well known as stimulators of bone formation. Both BMP4 and BMP6 have been shown to induce endochondral osteogenesis in animals and in vitro [20–22]. However, there is no evidence in vivo for the roles of BMP4 and BMP6 in HO of FOP. Based on all of the results mentioned above, we consider that the roles of BMP4 and BMP6 remain elusive at this time point, and their roles need to be investigated further.

In many human diseases, such as endocrine diseases, the blood levels of disease-causing factors (hormones) are abnormal. Therefore, we wondered whether there are abnormalities in the serum levels of activin A, BMP4 and BMP6 in FOP patients, either in the flare-up or remission period. To answer this question, we measured the serum activin A, BMP4 and BMP6 levels in 16 untreated FOP patients and 16 age- and sex-matched healthy control subjects. This study helps us to further reveal the disease profile of FOP.

## Methods

### Subjects

The present investigation was a cross-sectional observational study. A total of 16 untreated FOP patients and 16 age- and sex- matched healthy control subjects were recruited for the study between December 2015 and January 2016 at Tongji Hospital in Shanghai, China. All recruited subjects were Han people from China. FOP was diagnosed based on the classic clinical manifestations and genetic analyses [1, 23]. The clinical features of FOP include: (1) congenital malformation of the great toes; and (2) soft tissue swelling and at least one site of heterotopic ossification documented by X-ray film or CT scan. The flare-up was defined by the presence of at least two of six of the following symptoms: soft tissue swelling, pain, decreased range of motion, stiffness, redness, and warmth, which lasted for at least 2 days [24]. Remission was defined as at least 6 months free of flare-ups, and the patient state remained stable. The FOP patients were divided into 2 groups: (1) the flare-up group (*n* = 8), in which the patients had flare-ups or worsening of any joint movement in the last 6 months; and (2) the remission group (*n* = 8), in which the patients had no flare-up or worsening of joint movement in the last 6 months. Fasting venous blood samples were collected from all participants and processed within 30 min after collection. Written informed consent was obtained from all participants, and all human studies were approved by the Ethics Committee for Clinical Research of Tongji Hospital of Tongji University. The approval number was K-2013–009.

## Methods

Patient demographics, such as sex, age, clinical manifestations, family history and significant medical history, were collected. Genomic DNA was extracted from EDTA-treated blood samples according to standard protocols. ACVR1 genotyping was performed in all the studied subjects. To determine the activin A, BMP4 and BMP6 levels in plasma samples from the subjects, we used the activin A, BMP4 and BMP6 human enzyme‐linked immunosorbent assay (ELISA) Kit (Cloud-Clone Corp., USA) according to the manufacturer's instructions using undiluted samples analysed in duplicate. Samples, standards, or controls were then added into the kit wells and bound to the immobilized (capture) antibody. The sandwich was formed by the addition of the second (detector) antibody, and a substrate solution was added that reacts with the enzyme-antibody-target complex to produce a measurable signal. The detection ranges were 12.35–1000 pg/mL for activin A, 31.2–2000 pg/mL for BMP4 and 15.6–1000 pg/mL for BMP6. The minimum detectable concentrations were 4.99 pg/mL for activin A, 12.3 pg/mL for BMP4 and 5.5 pg/mL for BMP6. The intra-assay coefficient of variance was 6.7%, and all the samples were measured in the same batch.

### Statistical analysis

Exploratory data analysis and Shapiro‒Wilk tests were performed to determine the normality of the data distribution. Data are expressed as the means with standard deviation for continuous variables, median (25–75th percentile) for nonnormally distributed variables, and counts and percentages for categorical variables. Continuous variables were compared between groups with independent samples *t* tests or Mann‒Whitney U tests, and categorical variables were compared with the chi-square test or Fisher’s exact test. Univariate and multivariate logistic regression analyses were performed. These analyses were performed using IBM SPSS Statistics 23 software. Tests were two-sided, and a *p* value less than 0.05 was considered statistically significant.

## Results

**Fig. 1:**
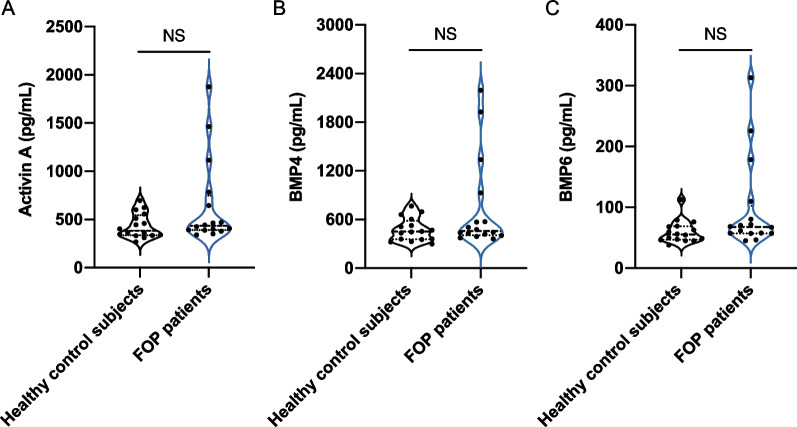
Serum activin A, BMP4 and BMP6 concentrations in healthy control subjects and FOP patients (NS: no significance)

Sixteen healthy subjects and sixteen FOP patients were enrolled in our study. The clinical characteristics of the subjects are shown in Table 1. The mean ages of the controls and the patients were 10.8 and 14.7 years, respectively. The sex ratio was also similar between the control group (37.5% for males) and FOP group (43.8% for males). Age and sex were comparable between the healthy control subjects and FOP patients (both *p* > 0.05). The median levels of serum activin A, BMP4 and BMP6 in healthy control subjects were 364.14 pg/mL, 450.39 pg/mL, and 55.36 pg/mL, respectively, while the levels of serum activin A, BMP4 and BMP6 in FOP patients were 434.05 pg/mL, 459.48 pg/mL, and 67.84 pg/mL, respectively. Although we observed slightly higher serum activin A, BMP4 and BMP6 levels in FOP patients, the differences between the two groups were not statistically significant (*p* > 0.05 for all) (Fig. 1).

**Table 1:** Clinical variables of all subjects

	Healthy control subjects (n = 16)	FOP patients (*n* = 16)	*z-*statistic	*p* value
Age (Y)	10.81 ± 6.99	14.72 ± 9.41	0.94	0.345
*Sex, No. (%)*				0.719
Male	6 (37.5)	7 (43.8)		
Female	10 (62.5)	9 (56.2)		
Activin A (pg/mL)	364.14 (335.29, 557.12)	434.05 (389.66, 748.63)	1.60	0.109
BMP4 (pg/mL)	450.39 (356.07, 583.34)	459.48 (406.64, 840.73)	1.11	0.266
BMP6 (pg/mL)	55.36 (46.72, 68.80)	67.84 (57.16, 102.50)	1.90	0.057

**Fig. 2:**
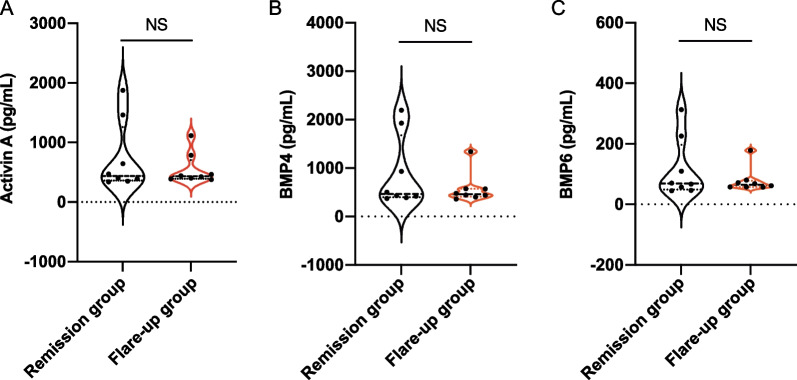
Serum activin A, BMP4 and BMP6 concentrations in FOP patients in remission and with flare-up (NS: no significance)

To further evaluate the roles of blood activin A, BMP4 and BMP6 in FOP flare-ups, the levels of serum activin A, BMP4 and BMP6 were compared between FOP patients in remission and those in the flare-up period. As shown in Table 2, in 8 of 16 (50%) FOP patients, the state remained stable, which is called “in remission”. The other 8 patients had a history of flare-ups in the last 6 months, which is called “in flare-up”. Patients in the remission group were slightly older than those in the flare-up group (18.93 vs. 10.49, *p* = 0.082). No statistically significant difference in sex was found between the two groups (*p* = 0.619). The median levels of serum activin A, BMP4 and BMP6 in the remission group were 438.48 pg/mL, 466.30 pg/mL and 68.80 pg/mL, respectively, while the levels in the flare-up group were 434.04 pg/mL, 459.48 pg/mL and 64.96 pg/mL, respectively. Notably, no significant differences in the concentrations of activin A, BMP4 or BMP6 were observed between the remission group and the flare-up group (*p* > 0.05 for all) (Fig. 2).

**Table 2:** Clinical variables of remission and flare-up FOP patients

Variable	Remission group (*n* = 8)	Flare-up group (*n* = 8)	*z-*statistic	*p* value
Age (Y)	18.93 ± 8.89	10.49 ± 8.37	1.74	0.082
*Sex, No. (%)*				0.619
Male	3 (37.50)	5 (62.50)		
Female	5 (62.50)	3 (37.50)		
Activin A (pg/mL)	438.48 (361.36, 1257.95)	434.04 (391.88, 703.68)	0.11	0.916
BMP4 (pg/mL)	466.30 (398.11, 1676.52)	459.48 (411.75, 571.98)	0.21	0.834
BMP6 (pg/mL)	68.80 (49.12, 196.91)	64.96 (57.64, 78.39)	0.05	0.958

To obtain more information about risk factors for FOP flare-up, we used univariate logistic regression analysis and multivariate logistic regression analysis for age, sex, serum activin A, BMP4 and BMP6 levels in FOP patients. The logistic regression analysis found no correlation between age, sex, serum activin A, BMP4 or BMP6 and the FOP flare-up (*p* > 0.05 for all) (Table 3).

**Table 3:** Univariate and multivariate logistic regression analyses for the risk of FOP flare-up

Variable	Univariate		Multivariate	
	OR (95%CI)	*p* Value	OR (95%CI)	*p* Value
Age	0.891 (0.783–1.015)	0.082	0.909 (0.777–1.063)	0.231
Sex	0.360 (0.048–2.725)	0.323	0.354 (0.026–4.800)	0.435
Activin A	0.989 (0.964–1.015)	0.399	1.070 (0.874–1.310)	0.513
BMP4	0.999 (0.997–1.001)	0.288	0.998 (0.989–1.008)	0.753
BMP6	0.992 (0.976–1.008)	0.338	0.966 (0.861–1.084)	0.561

## Discussion

In recent decades, numerous advances have been achieved in the field of FOP research, such as the proper characterization of disease progression through natural history studies, the identification of the causative gene and certain underlying molecular mechanisms, the development of in vitro and in vivo models resembling specific features of FOP, and the identification of specific cell types involved in HO and some important molecules with therapeutic potential. Despite these efforts, to date, there is no validated cure or biomarker for this catastrophic disease. Although activin A is a main pathogenic factor for FOP, are the levels of circulating activin A associated with the remission or flare-up of FOP? Are BMP4 and BMP6 as natural ligands of BMP signalling pathways associated with FOP?

Since activin A is the main pathogenic factor in FOP, we hypothesized that the levels of blood activin A may be increased in FOP patients. However, to our surprise, there were no significant differences in serum activin A concentrations between FOP patients and healthy control subjects, or between FOP patients with flare-up and those in remission. This suggests that blood activin A might not be the major pathogenic factor of FOP or a biomarker of flare-up in FOP patients. Activin A is a member of the TGF-β superfamily, which acts both in autocrine and paracrine manners as a hormone [25, 26]. Activin A regulates a host of important physiological and pathological processes locally and systemically, including immune and inflammatory responses, wound healing and fibrosis [27–31]. The serum concentration of activin A rapidly increases during inflammation. Activin A produced by inflammatory macrophages and other activated immune cells stimulates the release of inflammatory cytokines, such as tumour necrosis factor(TNF) and interleukin-1β(IL-1β), and promotes the recruitment of mast cells [29, 32–35]. Although the levels of blood activin A were significantly increased in other inflammatory diseases [36–38], this result was not observed in this FOP study. Activin A is expressed in many types of cells and tissues in human, such as endocrine cells of stomach, duodenum and pancreas [39], gallbladder [40] and kidney [41], etc., therefore, the local fluctuation of activin A expression does not necessarily change the blood level of activin A. Activin A can not only amplify dysregulated BMP pathway signalling through SMAD1/5/8 to induce endochondral bone formation of FOP, but also enhance the chondrogenic differentiation of progenitor cells through SMAD2/3 and promote injury-induced endochondral HO [17, 42]. The inhibition of activin A using antibodies could block HO in both FOP and acquired HO [9, 10, 42]. It is speculated that activin A plays a role in local FOP lesions in a paracrine or autocrine manner. It remains to be explored how activin A is expressed in local lesions upon injury and in spontaneous inflammation and whether local activin A can determine the pathological stages of FOP.

It is well known that FOP results from mutations in the intracellular domain of ACVR1, which displays neofunctional responses to activin A and is hyperactive to BMPs [9, 10, 19]. Hatsell et al. [9] generated HEK293/BRE-Luc reporter lines stably expressing either ACVR1 or ACVR1^R206H^ and tested their responses to a panel of ligands belonging to the BMP and TGF-β families. ACVR1^R206H^ displayed increased signalling in response to BMP4, whereas the response to BMP6 remained unchanged. In addition, Hino et al. [10] generated induced mesenchymal stromal cells from FOP patient-derived induced pluripotent stem cells (FOP-iMSCs) and mutation-rescued FOP-iMSCs (resFOP-iMSCs). These cells were transfected with a BMP-specific luciferase reporter construct and treated with TGF-β superfamily ligands. The results showed that several BMP ligands, such as BMP6, BMP7 and BMP4, induced higher luminescence in FOP-iMSCs than in resFOP-iMSCs. Culbert presented that BMP4 enhanced chondrogenesis of ALK2^R206H/+^ cells in vitro coupled with their induction of robust heterotopic endochondral ossification in vivo [22]. Importantly, BMP4 is highly expressed in injured muscle tissue and in inflammatory and fibroproliferative cells from early human FOP lesions [15, 43]. In addition, BMP6, as the natural ligand of ACVR1, could induce chondrogenesis through pSMAD1/5/8 [42]. ACVR1^R206H^ shows a higher sensitivity towards BMP6 than ACVR1^WT^ in vitro [19]. However, we found that there were no significant increases in serum BMP4 and BMP6 concentrations in FOP patients either in remission or in flare-up. These results suggest that blood BMP4 and BMP6 may not be pathogenic factors of FOP or biomarkers of flare-up in FOP patients.

### Study limitations

The present study has some limitations that warrant consideration. (1) FOP is an ultrarare disorder; therefore, the sample size of this study is small. (2) Such previous studies are scarcely reported, so we had few literature with which to compare our study. (3) The biopsy of lesions cannot be allowed by ethical regulations because it may induce HO at the surgical site, so we could not verify the protein expression of activin A, BMP4 and BMP6 in the local cells and tissue fluid of the lesion. (4) As this was a cross-sectional comparison study, future longitudinal follow-up studies are needed to address whether changes in the blood levels of activin A, BMP4 and BMP6 track with HO in FOP patients.

## Conclusions

In conclusion, this study compared the serum activin A, BMP4 and BMP6 levels between FOP patients (either in flare-up or in remission) and healthy control subjects. To our knowledge, this is the first report of such results. There were no significant changes in the serum levels of activin A, BMP4 or BMP6 in FOP patients, whether during periods of flare-up or during remission. Serum activin A, BMP4 and BMP6 may not be the causes of FOP flare-up and may not be used as potential biomarkers for FOP flare-up.

## Data Availability

All patient data has been anonymized, and any further information may be obtained from the corresponding author on reasonable request.

## Abbreviations

ACVR1: Activin A receptor type 1
ALK2: Activin receptor-like kinase 2
BMP: Bone morphogenetic protein
BMP4: Bone morphogenetic protein 4
BMP6: Bone morphogenetic protein 6
ELISA: Enzyme‐linked immunosorbent assay
FOP: Fibrodysplasia ossificans progressiva
HO: Heterotopic ossification
NS: No significance
PBMCs: Peripheral blood mononuclear cells
